# *Bacteroides thetaiotaomicron* enhances oxidative stress tolerance through rhamnose-dependent mechanisms

**DOI:** 10.3389/fmicb.2024.1505218

**Published:** 2024-12-11

**Authors:** Shuo Xie, Junze Ma, Zheng Lu

**Affiliations:** ^1^Hainan Province Key Laboratory of One Health, Collaborative Innovation Center of One Health, School of Life and Health Sciences, Hainan University, Haikou, Hainan, China; ^2^Guangdong Provincial Key Laboratory of Marine Biotechnology, Department of Biology, Institute of Marine Sciences, Shantou University, Shantou, China

**Keywords:** *Bacteroides*, rhamnose, SCFA, oxidative stress, PFOR

## Abstract

This study probes into the unique metabolic responses of *Bacteroides thetaiotaomicron* (*B. thetaiotaomicron*), a key player in the gut microbiota, when it metabolizes rhamnose rather than typical carbohydrates. Known for its predominant role in the Bacteroidetes phylum, *B. thetaiotaomicron* efficiently breaks down poly- and mono-saccharides into beneficial short-chain fatty acids (SCFAs), crucial for both host health and microbial ecology balance. Our research focused on how this bacterium’s SCFA production differ when utilizing various monosaccharides, with an emphasis on the oxidative stress responses triggered by rhamnose consumption. Notably, rhamnose use results in unique metabolic byproducts, including substantial quantities of 1,2-propanediol, which differs significantly from those produced during glucose metabolism. Our research reveals that rhamnose consumption is associated with a reduction in reactive oxygen species (ROS), signifying improved resistance to oxidative stress compared to other sugars. This effect is attributed to specific gene expressions within the rhamnose metabolic pathway. Notably, overexpression of the rhamnose metabolism regulator RhaR in *B. thetaiotaomicron* enhances its survival in oxygen-rich conditions by reducing hydrogen peroxide production. This reduction is linked to decreased expression of pyruvate:ferredoxin oxidoreductase (PFOR). In contrast, experiments with a *rhaR*-deficient strain demonstrated that the absence of RhaR causes *B. thetaiotaomicron* cells growing on rhamnose to produce ROS at rates comparable to cells grown on glucose, therefore, losing their advantage in oxidative resistance. Concurrently, the expression of PFOR is no longer suppressed. These results indicate that when *B. thetaiotaomicron* is cultured in a rhamnose-based medium, RhaR can restrain the expression of PFOR. Although PFOR is not a primary contributor to intracellular ROS production, its sufficient inhibition does reduce ROS levels to certain extent, consequently improving the bacterium’s resistance to oxidative stress. It highlights the metabolic flexibility and robustness of microbes in handling diverse metabolic challenges and oxidative stress in gut niches through the consumption of alternative carbohydrates.

## Introduction

The phylum Bacteroidota, formerly known as Bacteroidetes, predominantly populating the gastrointestinal tract of mammals, together with Bacillota (referred to as Firmicutes), comprises about 90% of the intestine’s total bacterial population (Patterson et al., 2016; Tajkarimi and Wexler, 2017; Illiano et al., 2020). Extensively studied, the genus *Bacteroides* within this phylum plays a crucial role in degrading complex carbohydrates that are otherwise indigestible for the host. This breakdown of dietary fiber leads to the fermentation process in the gut, resulting in the production of short-chain fatty acids (SCFAs) such as acetate and propionate (Rangan and Hang, 2017; Porter et al., 2018; Fang et al., 2019; Lapébie et al., 2019). These SCFAs are vital for the host, serving as an energy source after their transportation to the colon wall, passive diffusion into the bloodstream, and eventual uptake by various organs. Additionally, SCFAs contribute to a balanced gut microbiota by promoting the growth of beneficial bacteria and inhibiting the proliferation of harmful pathogens (Martin-Gallausiaux et al., 2021; Ye et al., 2021).

The genus Bacteroides encompasses various species such as *Bacteroides fragilis* (*B. fragilis*), *Bacteroides thetaiotaomicron* (*B. thetaiotaomicron*), *Bacteroides uniformis*, and *Phocaeicola vulgatus* (previously known as *Bacteroides vulgatus*) (Salyers, 1984; Wexler, 2007). Among these, *B. thetaiotaomicron* stands out as one of the predominant species and is considered the typical strain of Bacteroides (Comstock and Coyne, 2003; Ye et al., 2021). It is distinguished by its unparalleled array of carbohydrate-active enzymes relative to almost all other sequenced strains (Comstock and Coyne, 2003; Ye et al., 2021). It contains 172 glycosyl hydrolases, 11 enzymes that degrade host-derived products, 163 outer membrane polysaccharide-binding proteins, and 20 sugar-specific transporters. Moreover, its uncomplicated nutritional requirements allow it to metabolize a variety of sugars as substrates for carbon sources and energy acquisition (McKee et al., 2021; Ye et al., 2021). *B. thetaiotaomicron* is recognized as a highly effective polysaccharide-degrading bacterium and is increasingly acknowledged as an excellent model for investigating the mechanisms of bacterial polysaccharide degradation (Bry et al., 1996; El Kaoutari et al., 2013; Lapébie et al., 2019).

*Bacteroides* are present in the mammalian gastrointestinal tract, where the immediate proximity of intestinal epithelial cells to blood vessels facilitates efficient oxygen (O_2_) delivery. As O_2_ moves away from the blood vessels and into the lumen of the gut, its levels gradually decrease due to metabolic processes and cellular consumption. Colonocytes consume significant amounts of O_2_ via mitochondrial respiration, which impedes O_2_ diffusion into the gut (Lee et al., 2022). The presence of facultative anaerobes such as *E. coli* in the lumen may further deplete the limited O_2_, creating an almost anaerobic environment (Espey, 2013; Kelly and Colgan, 2016; Schwerdtfegerid et al., 2019; Bossuet-Greif et al., 2023; Lee et al., 2022). As a consequence, over 90% of enteric bacteria adopt strict anaerobic survival strategies. *Bacteroides* are among these obligate anaerobes, with *B. thetaiotaomicron* and *B. fragilis* commonly used as models for studying the anaerobic mechanisms of gut microbes (Pan and Imlay, 2001; Sund et al., 2008; Reott et al., 2009; Ito et al., 2020; Khademian and Imlay, 2020; Shin et al., 2024). They have developed fundamental oxidative defense mechanisms to counteract harmful reactive oxygen species (ROS) generated inside the cells, such as synthesizing antioxidant enzymes like superoxide dismutase (SOD), catalase and peroxidases (McCord et al., 1971; Gregory et al., 1978; Rocha et al., 2003; Mishra and Imlay, 2013; Lin et al., 2022). However, when exposed to aerobic environments, the elevated levels of endogenous ROS and O_2_ molecules can still damage crucial enzymes involved in energy metabolism. This disruption can lead to the breakdown of energy production, causing the bacteria to lose their energy source and cease proliferation (Pan and Imlay, 2001; Lu and Imlay, 2017; Lu et al., 2018).

In this context, rhamnose, a deoxy sugar commonly found in the glycans’ structures within the food matrix, transcends its basic role as a mere source of carbon and energy for enteric bacteria. The study suggests that its utilization by *B. thetaiotaomicron* may be linked to the bacterium’s ability to manage oxidative stress.

## Results

### Sugar utilization by *B. thetaiotaomicron* in relation to monosaccharides and their derivatives

While extensive research has examined the utilization of polysaccharides by *Bacteroides* spp., details on their specific preferences for certain monosaccharides remain scarce. This study explores the growth capabilities of *B. thetaiotaomicron* when cultured with nine diverse simple sugars and their derivatives as the sole carbon source.

**Figure 1:**
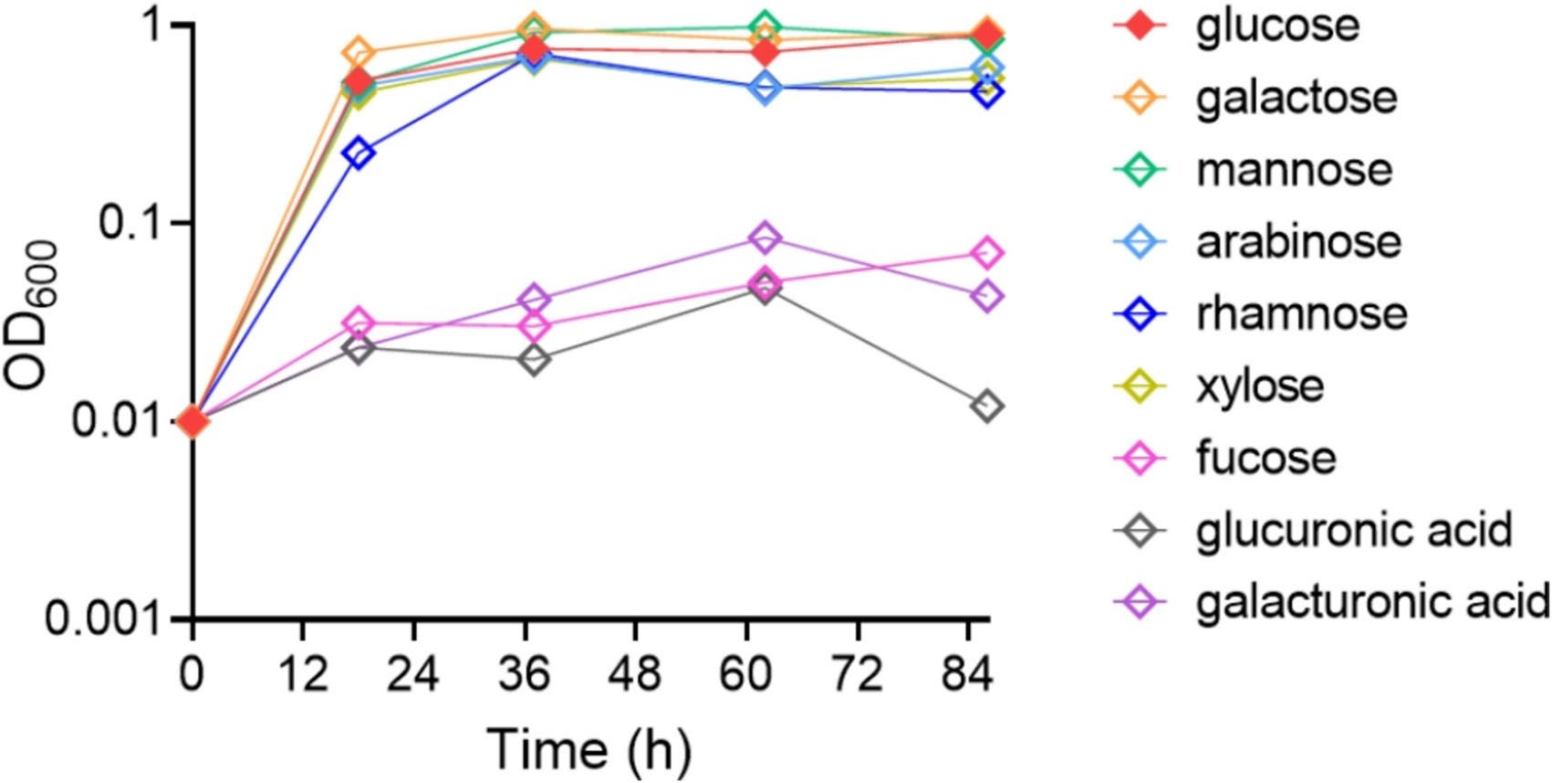
Growth dynamics of *Bacteroides thetaiotaomicron* with various monosaccharides as carbon sources. The growth curve data represent the average values from three independent experiments.

Bacterial growth was measured in the presence of different carbohydrates. The results indicate that *B. thetaiotaomicron* is capable of efficiently metabolizing most of the tested monosaccharides. Specifically, glucose, galactose, mannose, arabinose, rhamnose and xylose promoted rapid growth (Figure 1). The bacteria failed to sustain growth on fucose, glucuronic acid, and galacturonic acid when these were the only carbon sources provided.

We also simultaneously introduced glucose and rhamnose into the growth medium and monitored the consumption rates of these monosaccharides. Our observations indicated that *B. thetaiotaomicron* was concurrently utilizing both sugars as carbon sources for growth. Initially, glucose was consumed at a higher rate, but in the later stages of growth, the cells predominantly utilized rhamnose (Supplementary Figure S1).

### Quantify SCFAs produced by *B. thetaiotaomicron*

**Figure 2:**
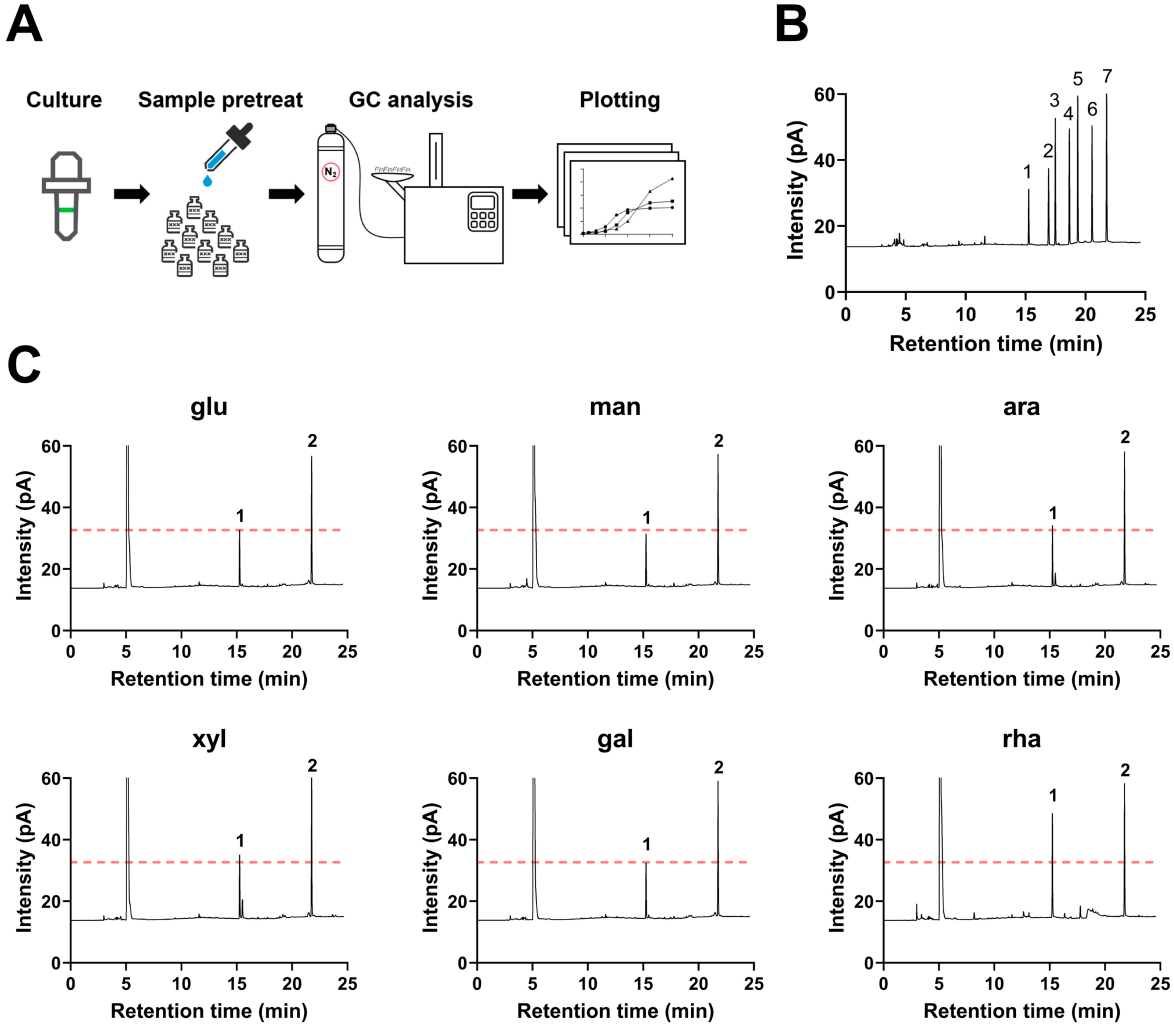
Gas chromatography (GC) detection of SCFAs produced by *B. thetaiotaomicron*. **(A)** Illustration of the SCFA quantification process via GC. **(B)** Retention times of SCFA standards. 1: acetic acid (15.26 min); 2: propionic acid (16.92 min); 3: isobutyric acid (17.48 min); 4: butyric acid (18.66 min); 5: isovaleric acid (19.36 min); 6: valeric acid (20.55 min); 7: 4-methylvaleric acid (internal standard). **(C)** GC peaks of principal metabolites from *B. thetaiotaomicron* after 48 h of growth. Peak 1: acetic acid; peak 2: 4-methylpentanoic acid (internal standard); leftmost peak represents methanol, which is added to the samples to stabilize them during gas chromatography and serves as a solvent carrier. For graphical clarity, only a portion of the methanol peak is shown. A red dashed line indicates the acetic acid peak when glucose is the carbon source. Labels: glu (glucose), man (mannose), ara (arabinose), xyl (xylose), gal (galactose), rha (rhamnose).

**Figure 3:**
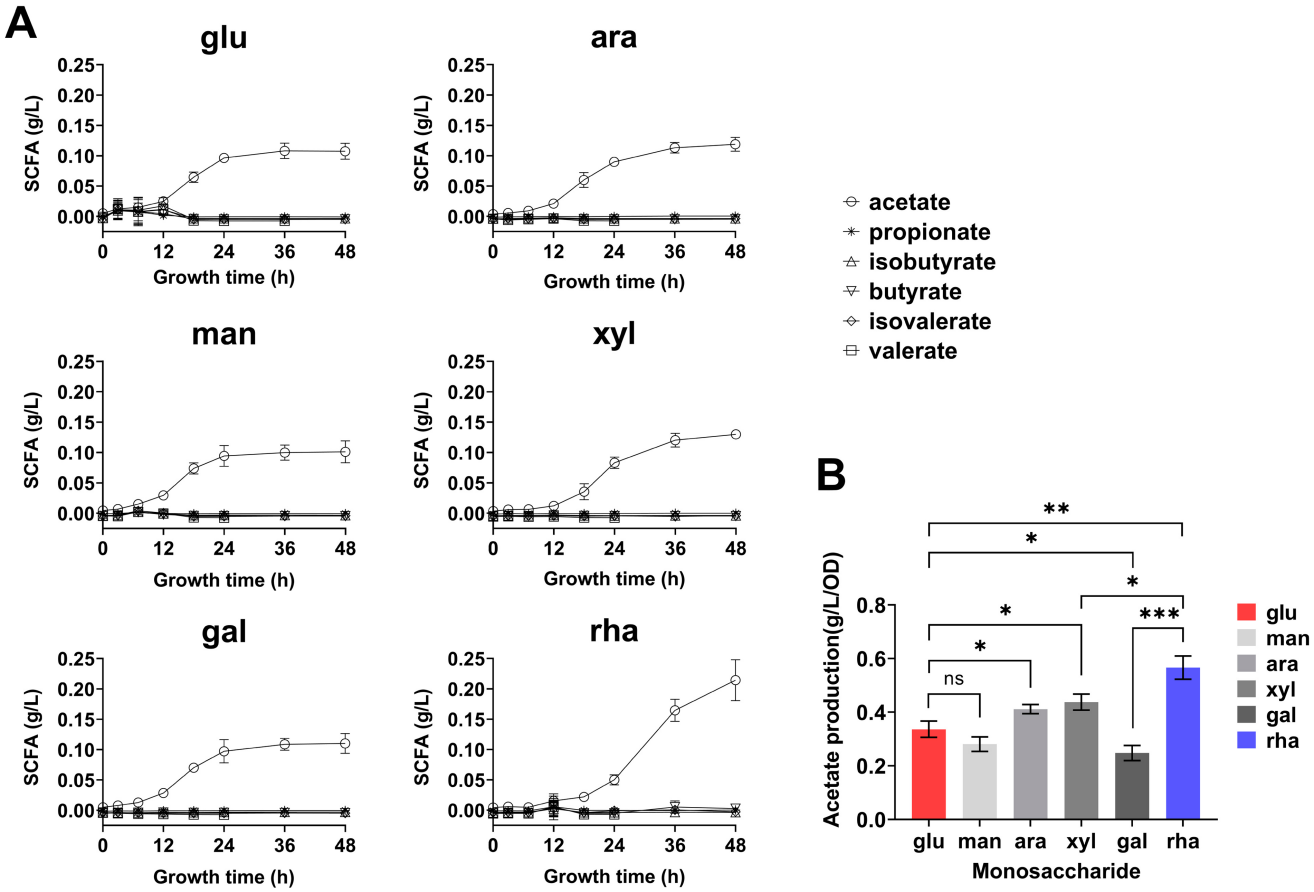
Short-chain fatty acids (SCFA) production by *B. thetaiotaomicron* during 48-h growth. **(A)** Real-time SCFA yield measured throughout a 48-h culture in DM media supplemented with various monosaccharides, using GC. **(B)** Acetic acid production rate calculated by the ratio of acetic acid at 48 h to cell density. Monosaccharides tested are labeled as in Figure 2. Error bars represent the SEM from at least three measurements. Statistical significance: ns (not significant), *p < 0.05, **p < 0.01, ***p < 0.001.

To explore the metabolic capability of *B. thetaiotaomicron* in producing SCFAs when cultivated on various monosaccharides as the sole carbon sources. *B. thetaiotaomicron* was cultivated using various monosaccharides as the sole carbon sources. During the culturing process, samples were collected every 4 h during the initial 12-h period, followed by collections every 6–12 h. For gas chromatography (GC) analysis, the samples were prepared according to the procedures outlined in the Materials and Methods section. The process diagram is illustrated in Figure 2A. The retention times of each SCFA were established by comparing them with standard substances, as shown in Figure 2B. The results indicated that *B. thetaiotaomicron* predominantly produced acetic acid, with negligible levels of other SCFAs such as propionic, isobutyric, butyric, valeric, and isovaleric acids detected throughout the fermentation course (Figures 2C, 3A,B). Notably, when grown in defined medium supplemented with rhamnose, referred to hereafter as DMR medium, *B. thetaiotaomicron* exhibited enhanced acetic acid production compared to other monosaccharides. Specifically, after 48 h of fermentation, the peak area of acetic acid from rhamnose was approximately twice that from other carbon sources, corresponding to an acetate concentration of 0.20 g/L (Figure 2C).

Over the period of a 48-h metabolic study, real-time GC analysis was performed to monitor the production of SCFAs by cells, again, no SCFAs other than acetic acid accumulated, and cellular growth in DMR medium was relatively slower compared to other defined media (Figure 3A). To normalize acetic acid production against cell density, the concentration of acetic acid was divided by the corresponding OD_600_ values. The results demonstrated that the production rate of acetic acid was markedly higher when rhamnose served as the carbon source, exceeding that observed with glucose by 1.6 times after 48 h (Figure 3B).

**Figure 4:**
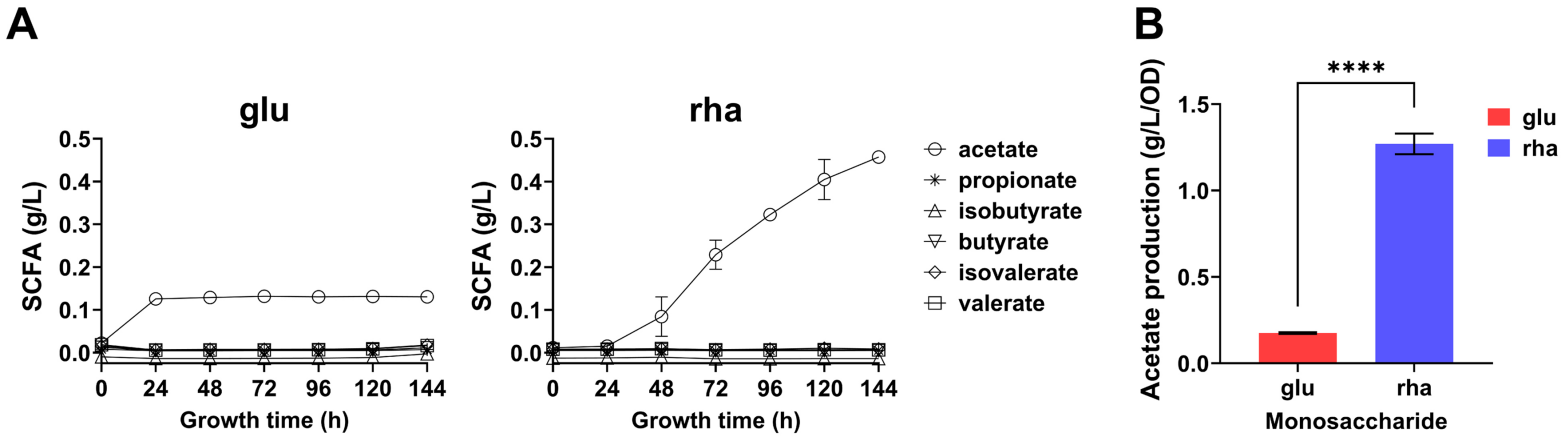
Six-day SCFA Production by *B. thetaiotaomicron*. **(A)** Real-time SCFA production rate. *B. thetaiotaomicron* was cultured in either DMG or DMR medium over 6 days, with periodic sampling for GC analysis of SCFAs. **(B)** Rate of acetate production on day 6; glu (glucose), rha (rhamnose). ****p < 0.0001. Error bars represent the SEM derived from at least three measurements.

*Bacteroides thetaiotaomicron* was cultured in DMR or DM medium supplemented with glucose (DMG) for 6 days, with daily sampling to monitor SCFA production. The results revealed that the acetic acid concentration in the DMR medium (0.45 g/L) was approximately 4 times higher than that in DMG after 6 days (Figure 4A). When acetic acid concentration from Day 6 was normalized to the culture’s OD_600_, the production rate from DMR was approximately 6 times higher than from DMG (Figure 4B).

During the analysis of cellular samples cultured in DMR medium for 6 days, GC monitoring revealed a peak corresponding to a non-SCFA compound, with an area comparable to that of acetic acid as indicated in Supplementary Figure S2. Notably, this peak was absent in cultures utilizing glucose or other monosaccharide carbon sources. Subsequent GC analysis identified the compound as 1,2-propanediol (Supplementary Figure S2). This finding is consistent with previous literature indicating that *B. thetaiotaomicron* metabolizes rhamnose via a phosphorylation-dependent metabolic pathway, leading to the production of propanediol (Patel et al., 2008; MacCabe et al., 2021). The pathway is distinct from the typical glycolytic breakdown of glucose, which primarily generates acetate, succinate and lactate (Pan and Imlay, 2001; Khademian and Imlay, 2020).

In addition to the peak for 1,2-propanediol, lactate was detected in samples cultured for 6 days (Supplementary Figure S2). Similar to propanediol, this peak was also absent in measurements taken during the 48-h culture period, suggesting that the accumulation of both lactate and propanediol within cells occurs more gradually than that of acetate. Despite these findings, another common fermentation byproduct, succinate, was not detected. This absence could be attributed to suboptimal GC detection conditions, indicating that adjustments to the GC column and vaporization temperatures may be necessary.

### *B. thetaiotaomicron* displays increased tolerance to oxidation when cultured in DMR medium

**Figure 5:**
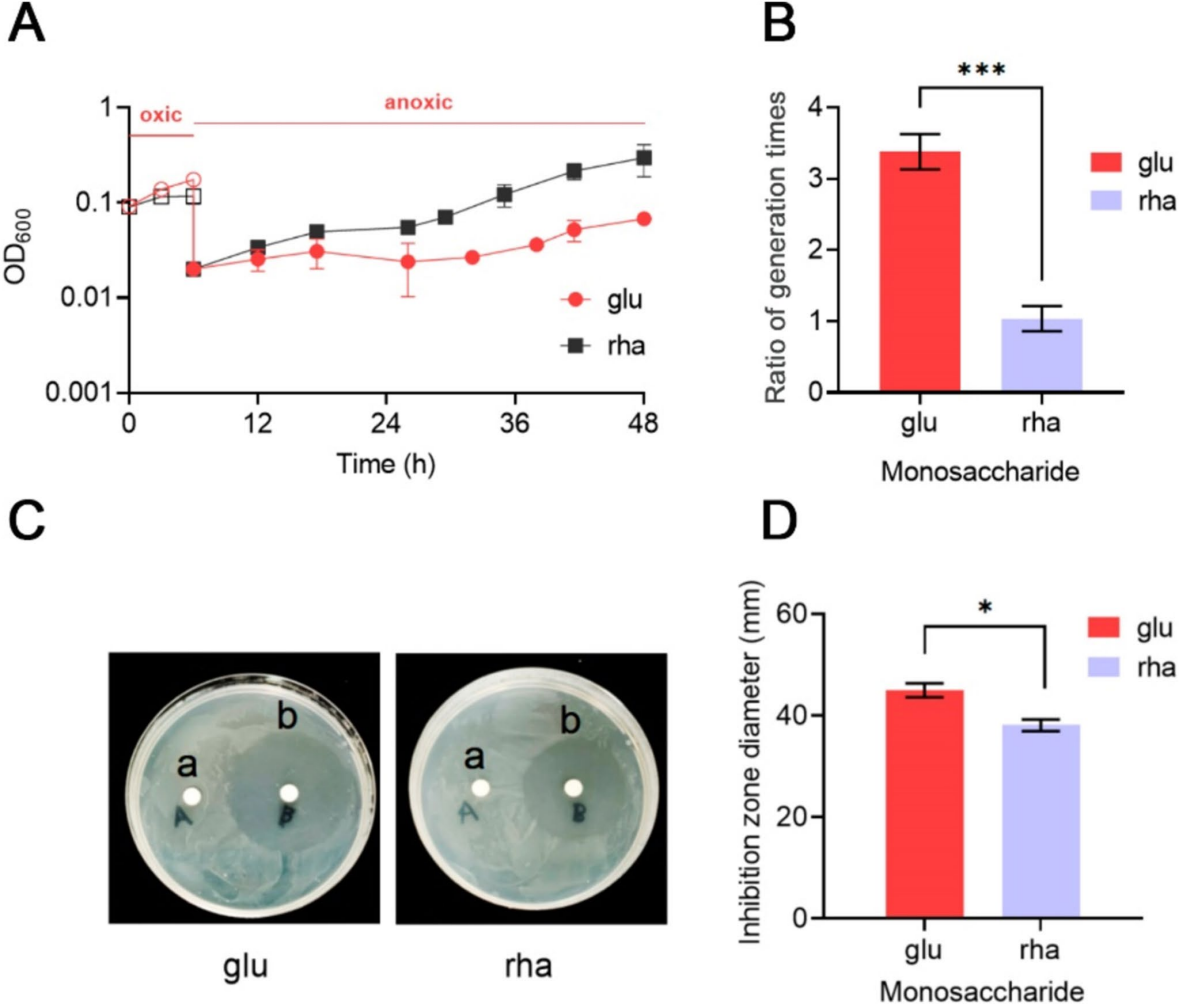
Impact of rhamnose metabolism on the growth of *B. thetaiotaomicron* under oxidative conditions. **(A)** Post-aeration recovery patterns of *B. thetaiotaomicron* cells after 6-h aeration. **(B)** Comparison of anaerobic growth generation times for *B. thetaiotaomicron* with and without exposure to air. **(C)** Inhibition zones caused by H_2_O_2_ on *B. thetaiotaomicron*. Cells were incubated on DM agar plates with either sterile water **(A)** or 1 M H_2_O_2_ **(B)** for 3 days, resulting in clear zones of complete bacterial inhibition. **(D)** Diameters of the inhibition zones. Cells were cultured in DM medium containing either glu (glucose) or rha (rhamnose) as the singular carbon source. *p < 0.05, ***p < 0.001. Error bars represent the SEM derived from at least three measurements.

*Bacteroides thetaiotaomicron*, a strictly anaerobic bacterium, is known for its high susceptibility to oxidative environments (Meehan et al., 2012; Lu and Imlay, 2017, 2021; Lu et al., 2018). This study examined its ability to resume growth after exposure to air. Initially, under anaerobic conditions in DMG medium, *B. thetaiotaomicron* displayed an average generation time of 3.5 h. Exposure to air completely inhibited growth, but subsequent reversion to anaerobic conditions allowed the bacterium to slowly restore its metabolic functions. However, its generation time extended to 3.38 times that of its normal rate (Figure 5A), with a noticeable increase in turbidity.

Growth experiments in DMR medium showed no growth under aerobic conditions. Yet, the difference in generation time after and before exposure to O_2_ was less marked, with a ratio of 1.07 (Figure 5B). These results indicate that *B. thetaiotaomicron*, when utilizing rhamnose as a carbon source, exhibits enhanced resistance to oxidative damages compared to glucose, facilitating quicker recovery from oxygen-induced stress.

*Bacteroides thetaiotaomicron*’s response to hydrogen peroxide (H_2_O_2_)-induced oxidative stress was further investigated using the agar diffusion method. In the immediate vicinity of the H_2_O_2_-soaked disks, a significant inhibition of *B. thetaiotaomicron* growth was observed, indicating substantial oxidative stress preventing normal bacterial metabolism and growth (Figure 5C). Notably, when cultured on DMR agar plates, which use rhamnose as the sole carbon source, the zone of inhibition measured approximately 38 mm in diameter. In contrast, on DMG plates, where glucose is the primary carbon source, the inhibition zone expanded to about 45.3 mm (Figure 5D). Despite the relatively minor variation in the inhibitory effect of H_2_O_2_ on *B. thetaiotaomicron* when using glucose and rhamnose as the sole carbon sources, repeated experimental validation confirmed the presence of this discrepancy. This finding indicates that *B. thetaiotaomicron* exhibits greater resilience to oxidative stress when grown in the presence of rhamnose compared to glucose.

### Regulation of rhamnose metabolism in *B. thetaiotaomicron* through RhaR overexpression

In our analysis, we examined the growth and metabolic features of *B. thetaiotaomicron* when utilizing l-rhamnose as a carbon source. The findings revealed a metabolic profile characterized by the production of acetic acid and 1,2-propanediol. Additionally, the strain displayed notable tolerance to oxidative environments. A question that arises is: How does the use of rhamnose contribute to the bacterium’s recovery and growth following oxidation exposure?

In *Bacteroides*, overexpression of the transcription factor RhaR can upregulate the overall transcription level of each gene of the *rhaKIPAO* gene cluster involved in rhamnose metabolism (Patel et al., 2009; Rodionova et al., 2013). We engineered a *rhaR* gene (BT3768) overexpression strain (named as using Bt-p*rhaR*) using the plasmid pNLY1-P*_susA_* (Shipman et al., 1999).

**Figure 6:**
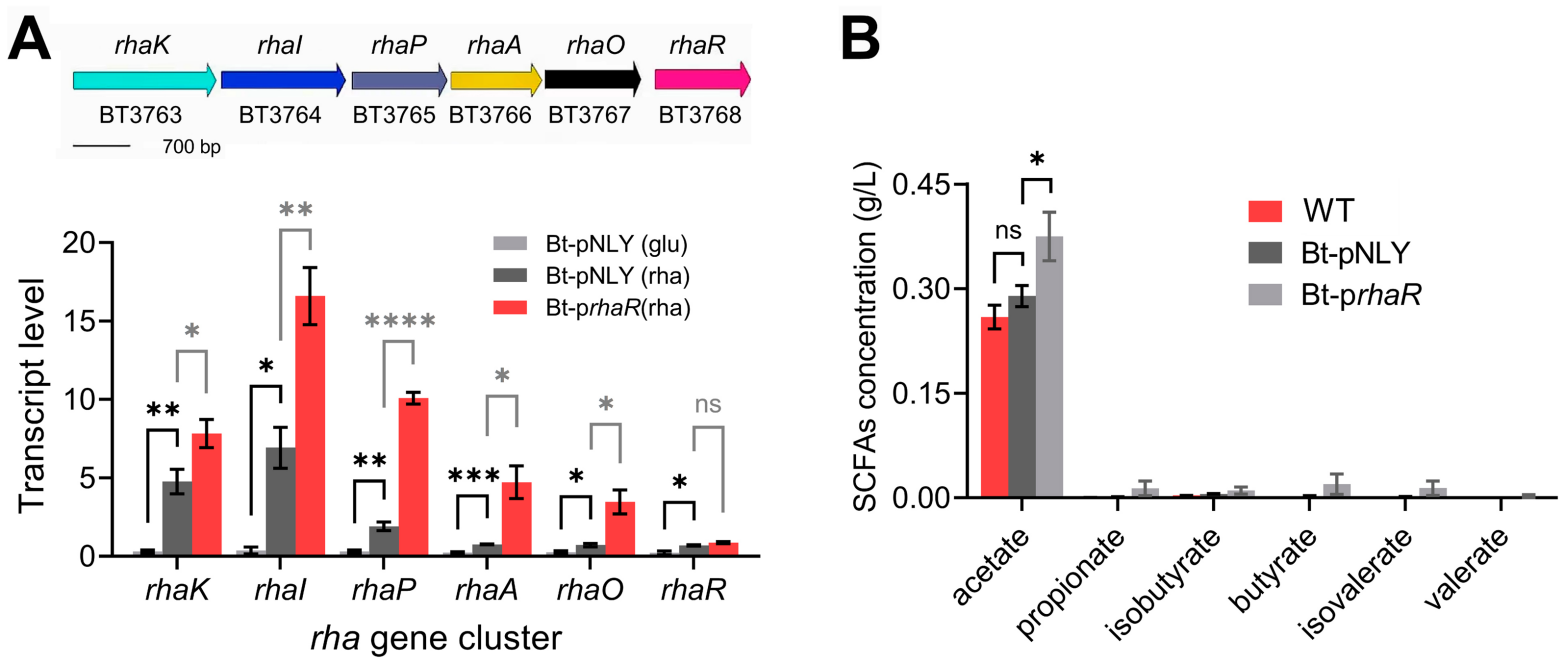
Overexpression of *rhaR* enhances rhamnose metabolism in *B. thetaiotaomicron*. **(A)** Diagram of *B. thetaiotaomicron* rhamnose metabolism gene cluster, including l-rhamnose permease (*rhaK*), isomerase (*rhaI*), kinase (*rhaP*), 1-phosphate aldolase (*rhaA*), lactaldehyde reductase (*rhaO*), and regulatory factor (*rhaR*). qPCR analysis on Bt-pNLY (with glucose “glu” or rhamnose “rha” as the carbon source) and Bt-p*rhaR* (rhamnose carbon source) strains, using 2^−ΔCt^ for absolute transcription levels. **(B)** Comparative SCFA production in *B. thetaiotaomicron* strains grown in DMR medium for 6 days, analyzed via GC. “ns” signifies no statistically significant difference, “*,” “**,” “***,” and “****” symbolize significance levels of *p* < 0.05, *p* < 0.01, *p* < 0.001, and *p* < 0.0001, respectively. Error bars represent the SEM derived from at least three measurements.

To verify the efficacy of the overexpression, RT-qPCR was employed to measure the transcription levels of the *rhaKIPAO* cluster in the modified strain. The results revealed significant upregulation of gene expression in Bt-p*rhaR* compared to a control strain harboring an empty vector (Bt-pNLY), particularly when grown in DMR medium (Figure 6A). Specifically, when Bt-p*rhaR* was cultured in DMR medium, the transcription levels of the *rhaKIPAO* gene cluster showed substantial increases with fold changes of 26.04, 44.37, 33.30, 19.98, and 13.40 respectively, compared to Bt-pNLY grown in DMG. When both strains were cultured in DMR medium, the relative transcription levels in Bt-p*rhaR* increased by fold changes of 1.64, 2.39, 5.27, 6.22, and 4.83 for each respective gene when compared to Bt-pNLY. Additionally, under rhamnose growth conditions, the *rhaR* expression levels in Bt-p*rhaR* exhibited a 3.84-fold increase compared to its expression in Bt-pNLY with glucose as the carbon source. Further comparative analysis also demonstrated that when Bt-pNLY utilized rhamnose instead of glucose, there was a notable enhancement in the transcription levels of genes within the *rhaKIPAO* cluster and *rhaR* itself, with fold changes of 15.85, 18.52, 6.31, 3.21, 2.77, and 3.05, respectively (Figure 6A). These findings indicate that *B. thetaiotaomicron*, when utilizing rhamnose as the sole carbon source, induces the *rhaKIPAO* cluster leading to commenced transcription. The overexpression of RhaR positively affects the transcription of the structural genes *KIPAO* within the *rha* operon.

Has the transcriptional upregulation of the *rhaKIPAO* cluster indeed enhanced rhamnose catabolism? This question was addressed by determining the metabolite production rates using GC analysis. To observe significant metabolite accumulation, cultures were incubated for 6 days. The results indicated that acetic acid production in the WT strain without a plasmid was comparable to that in the Bt-pNLY strain; however, production in the Bt-p*rhaR* strain was 32.4% higher than both (Figure 6B and Supplementary Figure S3). Notably, 1,2-propanediol was the primary non-fatty acid metabolite that significantly accumulated in all tested strains, while Bt-pNLY and the WT strain without plasmid showed similar synthesis rates for this metabolite, the Bt-p*rhaR* strain exhibited the highest production (Figure 6B). Furthermore, despite the overexpression of the RhaR protein, the yield of other SCFA products remained lower (Supplementary Figure S3).

### Rhamnose utilization benefits *B. thetaiotaomicron* cells to tolerate air exposure by reducing endogenous ROS generation

**Figure 7:**
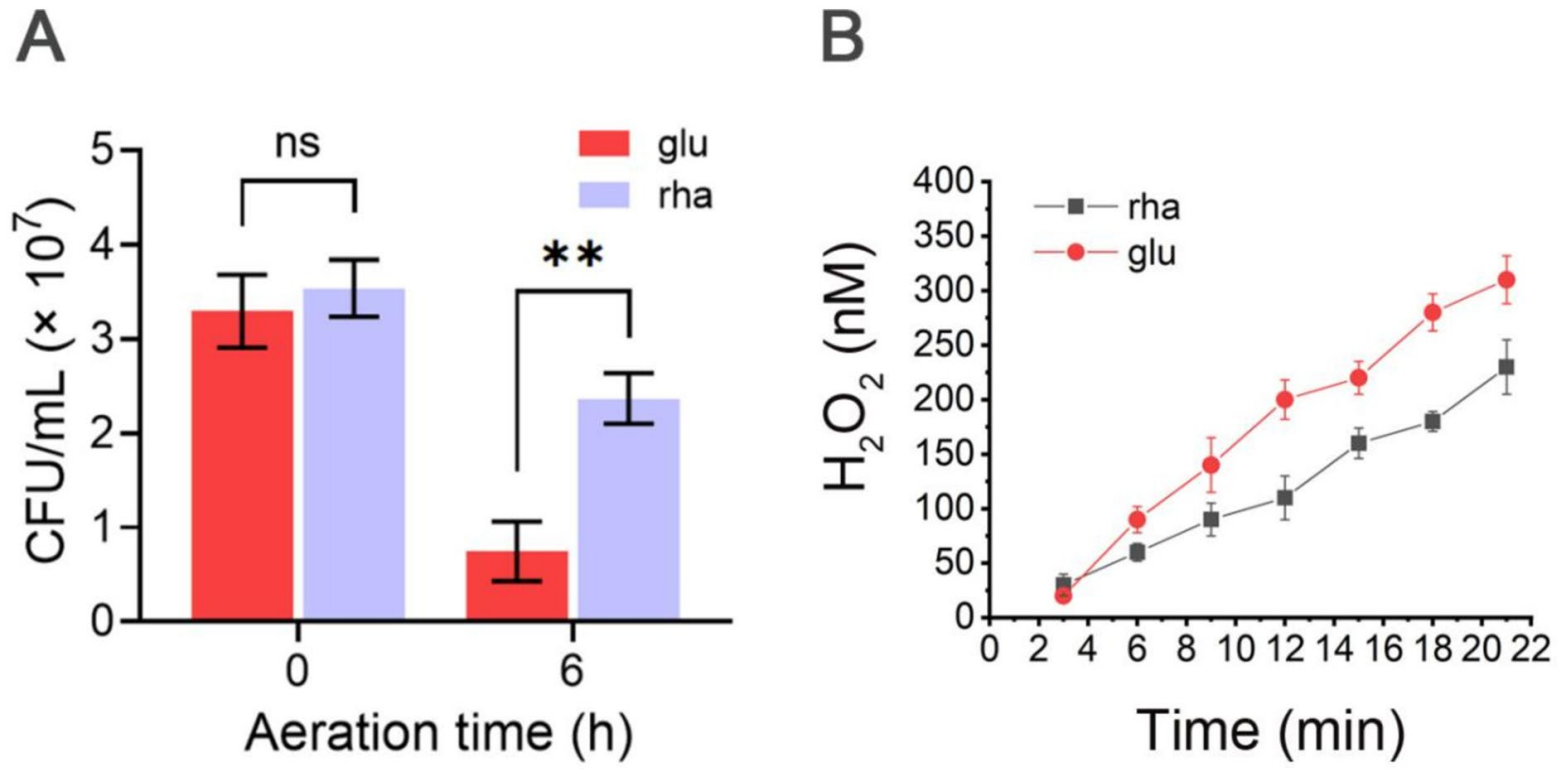
O_2_ tolerance and ROS mitigation via rhamnose metabolism in *B. thetaiotaomicron*. **(A)** Survival of Bt-p*rhaR* after aeration grown in DM medium with either glucose or rhamnose. Post-exposure to atmospheric O_2_ for 0 or 6 h, growth was assessed under anaerobic conditions. **(B)** H_2_O_2_ accumulation in SM136-p*rhaR*. Cells were anaerobically cultured in DMG and DMR media. The log-phase cells were re-suspended in oxygenated PBS (pH 7.2) plus either glucose (glu) or rhamnose (rha) at an OD_600_ of 0.01. Cell suspensions were sampled every 3 min, centrifuged to remove cells, and the resulting supernatant was analyzed for H_2_O_2_ concentrations using Amplex red dye. Detailed methodology is provided in Materials and methods. ns, not significant; **p < 0.01. Error bars represent the SEM derived from at least three measurements.

Bt-p*rhaR* cells were aerated for 6 h and survival was measured by comparing colony counts before and after air exposure. When grown with glucose as the sole carbon source, the survival rate of Bt-p*rhaR* after aeration was around 22%, while in the DMR medium, it reached 64.8% (Figure 7A). This result support the notion that using rhamnose as a carbon source for metabolism is more effective than glucose in shielding *B. thetaiotaomicron* from O_2_ toxicity.

Why can the use of rhamnose assist *B. thetaiotaomicron* cells in withstanding air exposure? This study seeks to understand whether it could help *B. thetaiotaomicron* cells manage ROS production and mitigate oxidative stress. To investigate this, we utilized a mutant strain of *B. thetaiotaomicron*, designated SM136, which was constructed by Imlay Lab (Mishra and Imlay, 2013) to lack major H_2_O_2_ degrading enzymes—specifically KatE, AhpCF, Rbr1, and Rbr2. Upon exposure to air, H_2_O_2_ is generated within the cells and subsequently diffuses into the extracellular space, allowing the measurement of extracellular H_2_O_2_ concentrations to infer internal ROS levels.

We further introduced either the p*rhaR* plasmid into the SM136 strain, resulting in the derivatives SM136-p*rhaR*. The Strain was cultured in DMG or DMR media to assess endogenous H_2_O_2_ production. Results indicated that SM136-p*rhaR* cells utilizing rhamnose produced H_2_O_2_ at approximately 11 nM/min, which was lower than the 16.5 nM/min observed in cells grown in DMG medium (Figure 7B). These findings support the hypothesis that rhamnose reduces ROS production in aerated *B. thetaiotaomicron* cells compared to glucose, thereby assisting the bacteria in managing oxidative stress.

### Why does rhamnose metabolism affect the production of H_2_O_2_?

Enhanced rhamnose metabolism has been shown to inhibit the activity of pyruvate:ferredoxin oxidoreductase (PFOR) (Patel et al., 2009), one of the enzymes responsible for converting pyruvate to acetyl-CoA in *B. thetaiotaomicron*, via alternative pathways. Such inhibition contributes to the increased resistance of *B. thetaiotaomicron* to metronidazole (Narikawa et al., 1991; Patel et al., 2009). In strains tolerant to metronidazole, PFOR transcription is notably reduced, suggesting that the diminished enzyme activity is due to lower transcriptional expression levels (Diniz, 2004). If PFOR activity is similarly inhibited in our experiments, it would restrict the electron flow through the enzyme, impacting the electron transfer to downstream low-redox potential components, which are considered as potential sources of intracellular ROS.

**Figure 8:**
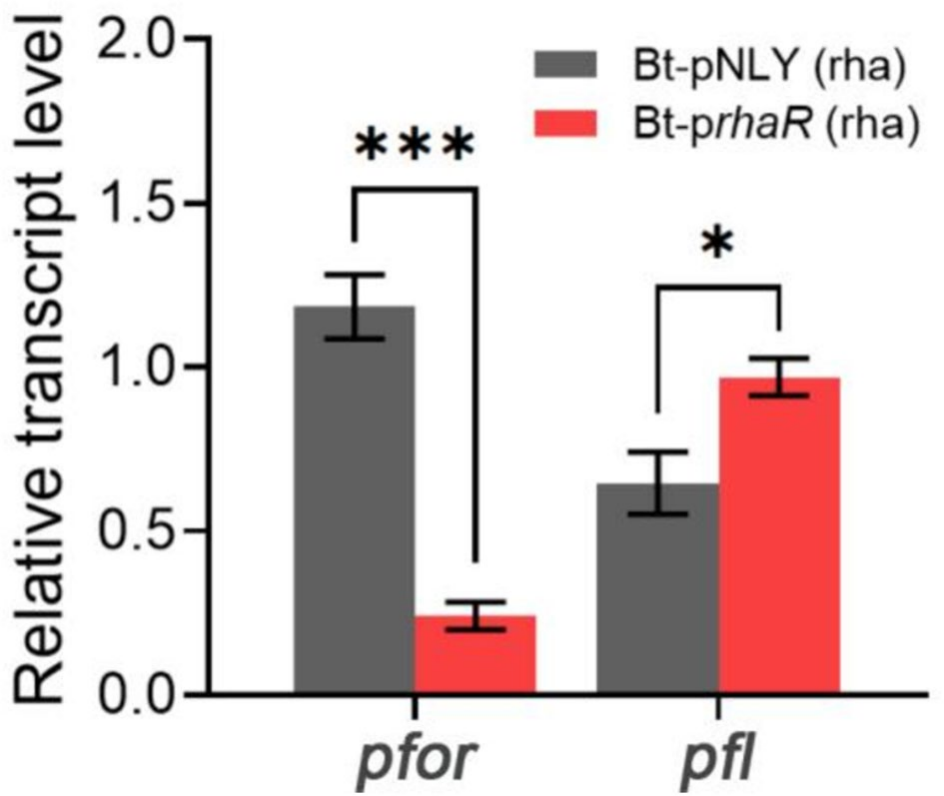
Effect of RhaR overexpression on PFOR and PFL gene transcription. Two groups of samples: Bt-pNLY and Bt-p*rhaR*, both fed with rhamnose as the carbon source. The relative transcription levels were calculated by 2^−ΔΔCt^ method, with the strain Bt-pNLY (glucose as the carbon source) serving as the control. *p < 0.05, ***p < 0.001. Error bars represent the SEM derived from at least three measurements.

RT-qPCR was employed to assess if the expression level of *pfor* was affected by the up-regulation of the rhamnose gene cluster. The results showed that the Bt-p*rhaR* strain, when grown in DMR medium, had a *pfor* gene transcription level that was substantially reduced to a fold change of 0.24 compared to the control Bt-pNLY strain, which utilized glucose (Figure 8). These findings suggest that elevated expression of the *rha* gene cluster suppresses the pyruvate oxidoreductase expression, thus reducing electron flow through the enzyme. Such inhibition could lead to diminished partial reduction of O_2_ molecules by downstream components, possibly decreasing ROS generation.

Pyruvate:formate lyase (PFL) serves as an alternative pyruvate-dissimilating enzyme that splits pyruvate into acetyl-CoA and formate. It offers a pathway for pyruvate dissimilation that is independent of PFOR, effectively circumventing the need for downstream electron transfer (Knappe et al., 1984; Wagner et al., 1992; Marquet et al., 2007). RT-qPCR was also used to determine whether the expression levels of PFL were affected by the overexpression of RhaR. The results indicated that the transcription level of PFL in the Bt-p*rhaR* strain, cultivated in DMR medium, was comparable to, or even slightly higher than, that observed in the Bt-pNLY strain grown in DMG (Figure 8).

### The transcription factor RhaR, rather than downstream rhamnose catabolism, confers oxidative stress tolerance on *B. thetaiotaomicron*

**Figure 9:**
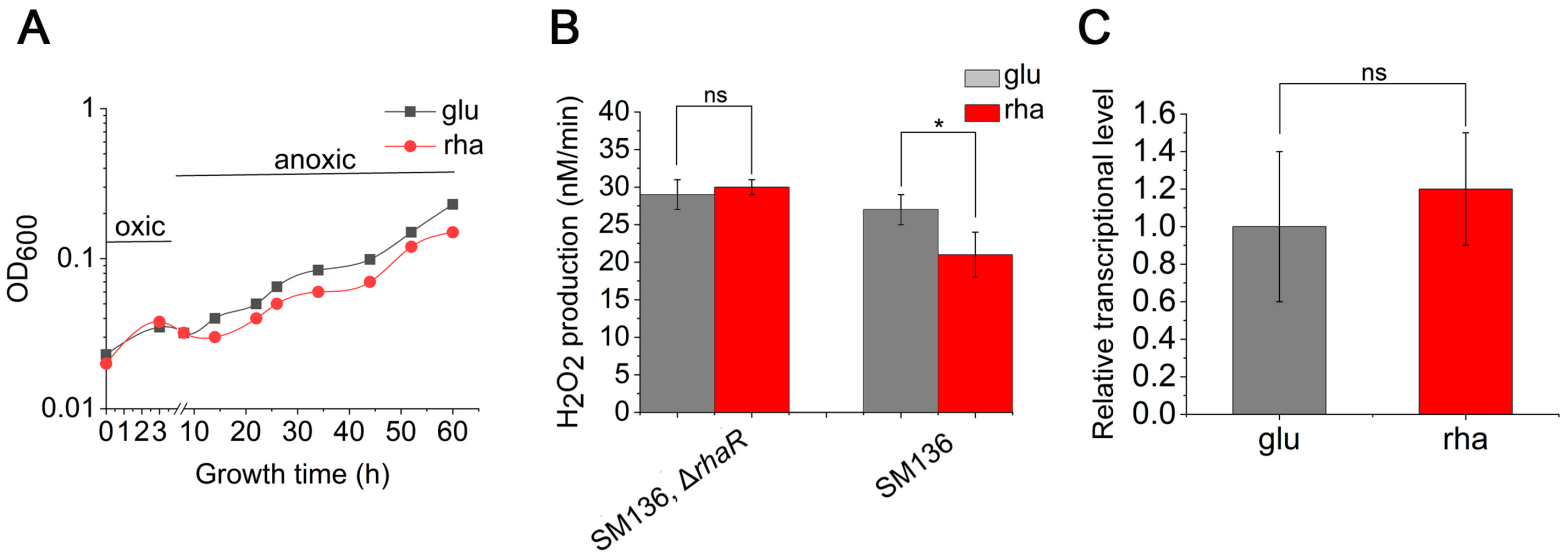
Deletion of *rhaR* impairs the oxidative tolerance of *B. thetaiotaomicron*. **(A)** Recovery growth of *rhaR* mutant following oxidative treatment in DMG (glu) and DMR (rha) anaerobic media. **(B)** Variation in H_2_O_2_ production rates in mutants grown in DMG and DMR media. **(C)** qPCR analysis comparing *pfor* expression in *rhaR* mutant across glucose (glu) and rhamnose (rha) carbon sources. *p < 0.05; ns, not significant. Error bars represent the SEM derived from at least three measurements.

The expression of *rhaR* positively influences the transcription of *KIPAO* operon, boosting rhamnose metabolism. It remains to be determined whether it is the regulator RhaR itself or the degradation process of rhamnose that aids *B. thetaiotaomicron* in tolerating O_2_. To investigate this, the *rhaR* gene was knocked out in the SM136 strain, creating a mutant designated as JZ005, and subsequent assays were performed. Despite the absence of RhaR, JZ005 still consumed rhamnose as the carbon source to grow (Supplementary Figure S4). The test for anaerobic growth recovery following hyperoxia exposure showed no discernible difference in the growth of JZ005 between DMG and DMR media (Figure 9A). H_2_O_2_ production measurements demonstrated that the rates of H_2_O_2_ production in JZ005 were consistent during cell growth under DMG and DMR culture conditions (Figure 9B). Further qPCR verification confirmed that, in the Δ*rhaR* background, the expression level of *pfor* remained unchanged when the carbon source was switched from glucose to rhamnose (Figure 9C). These findings demonstrate that the absence of *rhaR* may weaken the cellular ability to catabolize rhamnose, yet it does not affect the expression of *pfor* in response to shifts in carbon sources, nor does it impact the aerobic tolerance of *B. thetaiotaomicron* across different sugar substrates.

### Rhamnose has a modest role in activating cellular oxidative defenses

In *B. thetaiotaomicron*, rhamnose metabolism may be linked to specific cellular processes that aid in combating oxidative stress by potentially boosting the expression of antioxidant enzymes. To investigate this hypothesis, we assessed the impact of rhamnose on the transcription levels of these antioxidants in *B. thetaiotaomicron* cells. The enzymes involved in degrading H_2_O_2_ in *B. thetaiotaomicron* include AhpCF, KatE, Rbr1, and Rbr2 (Mishra and Imlay, 2013). SOD catalyzes the dismutation of superoxide radicals (O_2_^−^) into hydrogen peroxide (H_2_O_2_), thereby mitigating oxidative stress by facilitating the removal of these radicals. To introduce oxidative damages, cells were exposed to air for 1.5 h, sufficient to trigger a response (Mishra and Imlay, 2013). Gene transcription changes were quantified by qPCR before and after air exposure.

The results revealed that overexpression of RhaR in bacteria, with rhamnose as the carbon source, led to a modest increase in the transcription of the *rbr2* gene, showing a fold change of 1.53 recorded after aeration versus before (Supplementary Figure S5). Rhamnose did not significantly influence the transcriptional expression of other ROS defense enzyme genes, including *katE, ahpCF, rbr1,* and *sod* (Supplementary Figure S5).

## Discussion

In this study, we demonstrated that that *B. thetaiotaomicron*, when exposed to air, can employ rhamnose to reduce intracellular ROS production, thereby enhancing its capacity to withstand oxidative pressure. Unlike glucose metabolism, the degradation of rhamnose uniquely results in the substantial synthesis of acetic acid and 1,2-propanediol, highlighting the distinctive pathways involved in rhamnose utilization as compared to glucose. The findings here indicate that the intestinal anaerobe *B. thetaiotaomicron* can efficiently alleviate oxidative stress damage and boost SCFAs production through rhamnose utilization. It may deepen our understanding of the adaptive strategies and physiological regulatory mechanisms of these bacteria in dynamic environments, contributing to the expansion of knowledge about the intricate interactions between intestinal microbiota and their hosts.

### Potential adaptation mechanisms of gut anaerobes in response to oxidative stress

Strict anaerobes struggle to survive in O_2_-rich environments for various reasons, and the exact mechanisms behind this are still unclear. Evidences suggest that certain bacteria may adapt to oxidative environments by metabolizing specific nutrients (Li et al., 2022; Santamaria et al., 2022). This adaptability is vital for species like *Bacteroides* spp., which reside in the gut microbiota—a dynamic ecosystem where O_2_ levels can vary due to factors such as inflammation, dietary shifts, or the activity of other microbes that either consume or produce O_2_ (Kelly and Colgan, 2016; Schwerdtfegerid et al., 2019; Huang and Li, 2020). It’s possible that enteric anaerobes may switch to using specific metabolic processes to cope with temporary oxidative stresses. Such metabolic flexibility could have significant implications for human health. Therefore, understanding the survival mechanisms of gut bacteria during intermittent O_2_ exposure can not only expand our knowledge of microbial physiology and adaptive strategies but also highlight the interconnectedness of microbial health and human well-being, paving the way for new therapeutic avenues and preventive measures.

The detailed study of *B. thetaiotaomicron*’s carbohydrate metabolism underscores its role as a versatile organism within the gut microbiota, proficient at utilizing a diverse array of nutrient sources to flourish in varied niches within the gastrointestinal tract. This trait may not only help in its own survival but also influence the overall composition of the gut microbiome, promoting a balanced microbial ecology. *B. thetaiotaomicron* exhibits robust catabolic activity across a range of monosaccharides (Figure 1). Notably, slower growth rates when metabolizing rhamnose compared to other monosaccharides suggest a more intricate metabolic pathway. Rhamnose, prevalent in the human gut (Flint et al., 2012; Sonnenburg and Sonnenburg, 2014), can be degraded anaerobically by enteric bacteria into SCFAs. When *B. thetaiotaomicron* catabolizes rhamnose, it primarily produces more acetic acid as SCFAs compared to other carbon sources. Acetic acid and other short-chain fatty acids (SCFAs) are crucial for maintaining colonic health. A deficiency in these metabolites may be associated with disorders such as inflammatory bowel disease (IBD), obesity, and diabetes (Louis et al., 2014; Martin-Gallausiaux et al., 2021).

### Rhamnose fermentation in *B. thetaiotaomicron*

**Figure 10:**
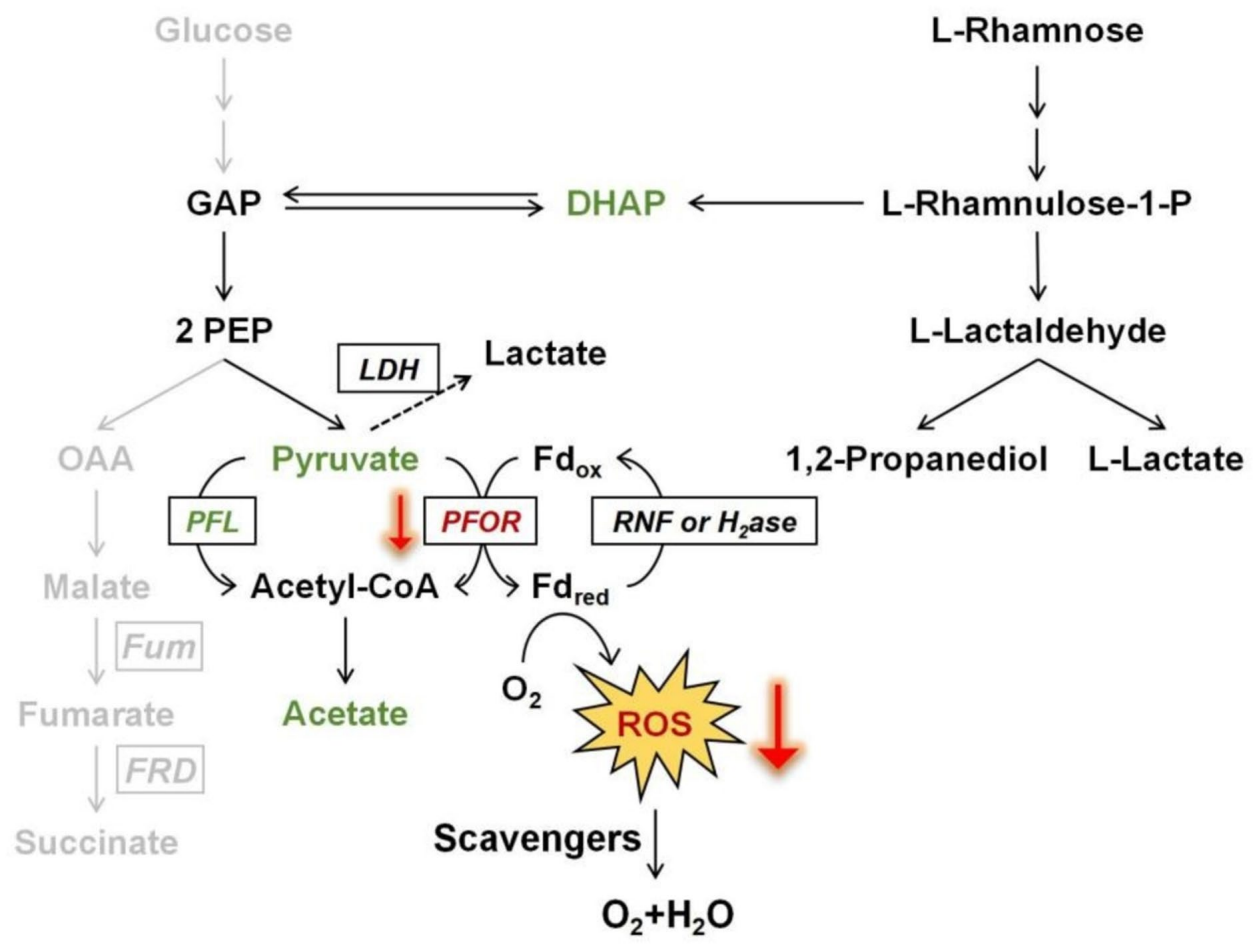
Scheme of promoting oxidative stress tolerance through rhamnose metabolism in *B. thetaiotaomicron.* The downward red arrows demonstrate that the transcriptional expression of PFOR is affected by rhamnose metabolism, which in turn influences the production of ROS at that node. The gray pathway illustrates a branch of glucose fermentation. Fd, ferredoxin; GAP, glyceraldehyde-3-phosphate; PEP, phosphoenolpyruvate; OAA, oxaloacetate; DHAP, dihydroxyacetone phosphate; PFL, pyruvate formate-lyase; PFOR, pyruvate: ferredoxin oxidoreductase; RNF, ferredoxin: NAD oxidoreductase; H_2_ase, hydrogenase; Fum, fumarase; FRD, fumarate reductase; LDH, lactate dehydrogenase.

During the fermentation of rhamnose by *B. thetaiotaomicron*, acetic acid and 1,2-propanediol are the primary byproducts (Figures 3, 4). How are these products produced? The metabolic pathway begins with the phosphorylation of rhamnose, leading to the production of dihydroxyacetone phosphate (DHAP) and l-lactaldehyde (Figure 10). l-lactaldehyde is then converted into 1,2-propanediol and l-lactic acid by lactaldehyde dehydrogenase (MacCabe et al., 2021). Following this, DHAP is transformed into glyceraldehyde-3-phosphate (GAP), which in turn can be transformed into pyruvate. Pyruvate is then decarboxylated, primarily by the enzymes PFOR and PFL, to produce acetic acid (Imlay et al., 2019; Khademian and Imlay, 2021). Research from Patel et al. (2009) illustrates that overexpression of the gene *rhaR* in *B. thetaiotaomicron* diminishes PFOR activity while escalating lactate dehydrogenase activity. This alteration likely leads to a reduction in PFOR’s involvement in acetic acid production, favoring acetyl-CoA synthesis via PFL. PFL cleaves pyruvate into acetyl-CoA and formate, with acetyl-CoA subsequently converted to acetic acid. Rhamnose metabolism, which involves a series of enzymatic steps more complex than those for simpler sugars such as glucose, appears to facilitate a pathway for rhamnose fermentation that produces acetic acid, as illustrated in Figure 10. This represents a metabolic branch not previously documented. Additionally, the novel contribution of our research is the finding that the expression of the regulator RhaR inhibits the transcription of *pfor*, with PFL compensating for this decrease. This investigation into compensatory mechanisms provides new insights into the metabolic regulation of *Bacteroides* across different carbon sources, as illustrated in Figure 8.

### Implications of rhamnose consumption on oxidative stress management

Is there a correlation between these findings and the elevated bacterial oxidative tolerance upon rhamnose utilization? Further investigations confirm that enhancing rhamnose metabolism correlates with a reduction in endogenous ROS production. Notably, in a mutant strain lacking major H_2_O_2_ scavenging enzymes, the overexpression of rhamnose degradation genes led to a diminished rate of H_2_O_2_ accumulation when cultured in DMR medium, compared to growth on glucose (Figure 7). The decrease in H_2_O_2_ levels might be linked to the downregulation of PFOR enzyme expression. ROS generation is hypothesized to arise from the autoxidation of enzymes located at the pyruvate node within central metabolism (Lu et al., 2018). Due to the high electron flux rate at this juncture, conditions may become favorable for ROS formation, particularly involving low-potential cofactors, may predispose to ROS formation. The other side, Figure 1 illustrates the relatively slow growth on rhamnose, which does not seem to impact the cells’ capacity to produce ROS. As depicted in Figure 9B, deletion of the *rhaR* gene indicates that ROS production rates in cells grown on a rhamnose-based medium are comparable to those in cells grown on glucose. This suggests that ROS production is primarily regulated by RhaR-mediated mechanisms. In the absence of RhaR, there is no down-regulation of PFOR expression, and consequently, cellular ROS levels remain unaffected.

It should be highlighted that PFOR is not the main source of intracellular ROS in *B. thetaiotaomicron*, as previously pointed out (Khademian and Imlay, 2020). However, we noticed that the rate of ROS production in cells with the PFOR gene knocked out was slightly lower than in the wild-type background (Khademian and Imlay, 2020). When its expression level is significantly affected, there can be a minor impact on ROS levels in the cells. Additionally, earlier studies have not assessed ROS production in *B. thetaiotaomicron* when rhamnose was the sole carbon source, a condition under which the role of PFOR in ROS production might differ.

The observation that a significant amount of acetic acid was produced and the expression level of *pfor* declined as cells catabolized rhamnose emphasizes an alternative route of pyruvate dissimilation dependent of PFL. PFL represents another mechanism for breaking down pyruvate without producing NADH, thereby helping to maintain cellular redox balance (Lu and Imlay, 2021). Under such conditions, ROS level did not increase, apparently the PFL-related metabolic pathway has a minimal impact on ROS formation.

Creating the *rhaR* deletion strain allows for the identification of whether it is the RhaR protein itself or the catabolism process of rhamnose that affects the intracellular ROS production levels. The abolishment of *rhaR* causes *B. thetaiotaomicron* to forfeit its tolerance advantage in O_2_-rich environments. This is primarily due to the deletion of *rhaR*, which leads to the expression of *pfor* no longer being influenced by the presence of rhamnose or glucose as carbon sources. As a result, the changes in the intracellular ROS levels exhibit minimal variation during cell growth in DMG and DMR media (Figure 9). These findings contribute new insights into how rhamnose utilization can mitigate ROS levels in *B. thetaiotaomicron*, thereby enhancing its tolerance to oxidative stress.

Besides, the metabolism of rhamnose marginally influenced the transcription of *rbr2* and did not significantly affect the expression of other ROS-scavenging enzymes, suggesting that rhamnose may have a minor impact on the activation of the ROS detoxifying system. This observation is corroborated by results from the H_2_O_2_ inhibition experiment, where *B. thetaiotaomicron* demonstrated slightly improved resistance to H_2_O_2_ during rhamnose metabolism compared to glucose, but the difference was not significant (Figure 7).

### Oxidative damages in anaerobic bacteria upon oxygen exposure

The oxidative damages experienced by strictly anaerobic bacteria due to air exposure is multifaceted, affecting not only PFOR but also key metabolic enzymes such as fumarase and PFL, as well as mononuclear iron enzymes involved in branched-chain amino acid synthesis (Khademian and Imlay, 2020; Lu et al., 2018). These enzymes are compromised in aerobic environments; PFOR and PFL are primarily inactivated directly by molecular oxygen, whereas enzymes like fumarase are mainly deactivated by ROS such as O_2_^−^ and H_2_O_2_, produced internally. The inactivation of PFOR and PFL by O_2_ is inevitable upon air exposure. However, the deactivation of other enzymes correlates with intracellular ROS levels; reduced ROS levels could decelerate damage to these enzymes and their metabolic functions, facilitating cellular recovery and growth. When rhamnose is used as the carbon source, we observed a reduction in the rate of ROS production within the cells, which alleviates the extent of damage to enzymes such as fumarase. In this paper, we currently do not further analyze whether the targets of oxidative damage vary when bacteria utilize different carbon sources and the physiological metabolic effects this may cause.

Overall, the experiments reported in this study demonstrate that the rhamnose utilization of *B. thetaiotaomicron* offers a defense mechanism against intermittent oxidative stress. The shift toward rhamnose consumption enhances the management of oxidative stress by decreasing H_2_O_2_ levels, coupled with notable downregulation of PFOR enzyme expression (Figure 10). This adjustment is mediated by the RhaR regulator within the rhamnose metabolic cluster. We still do not fully understand how RhaR affects the expression of *pfor*, as both are participants in rhamnose utilization and metabolism. The exact means through which each influences the expression of the other remains to be studied further. It is yet to be investigated whether RhaR directly binds to the promoter of the *pfor* gene or affects its expression indirectly through other intermediary molecules or signaling pathways. More investigations are needed in the future. The direct activation of ROS-detoxifying enzymes appears modest. The capacity of *B. thetaiotaomicron* to mitigate oxidative stress through specific metabolic pathways underscores the intricate interactions between gut microbes and their environments, highlighting potential therapeutic targets or dietary interventions that could harness these microbial mechanisms for improved health outcomes.

## Materials and methods

### Chemicals

Brain heart infusion (BHI) broth, LB broth, and LB agar from Huankai Microbial.; chemicals like l-cysteine hydrochloride and antibiotics from Yuanye Bio-technology; sugars and organic acids from Aladdin; other laboratory chemicals including hemin chloride and various organic acids from Macklin; basic laboratory reagents like glucose, agar powder, and acids from Xilong Scientific; specialized items like 30% hydrogen peroxide from Ghtech, agarose from Yeasen Biotechnology, and TAE Buffer from Sangon Biotech.

### Strains, plasmids, and growth conditions

Bacterial strains and plasmids are detailed in Supplementary Table S1. *B. thetaiotaomicron* was grown in either BHI or defined minimal medium (DM) (Bacic and Smith, 2008) with various carbon sources, depending on experimental requirements. These carbon sources included monosaccharides like d-glucuronic acid, d-galactose, d-galacturonic acid monohydrate, d-mannose, d-xylose, l-fucose, l-arabinose, l-rhamnose monohydrate, and sugar alcohols like sorbitol, erythritol, maltitol, xylitol, stevioside, aspartame, acesulfame, saccharin, sucralose and sodium cyclamate replaced glucose in DM. Media preparation involved autoclaving and overnight deoxygenation, followed by anaerobic culturing at 37°C with a specific gas mixture (85% N_2_, 10% H_2_, 5% CO_2_) and O_2_ levels under 50 ppm. *E. coli* was aerobically cultured in LB media at 37°C. Optional antibiotics (gentamicin 200 μg/mL, ampicillin 100 μg/mL, chloramphenicol 25 μg/mL) were added as needed.

### GC measurement of metabolites

*Bacteroides thetaiotaomicron* cells incubated under anaerobic conditions in BHI to the logarithmic phase (OD_600_ = 0.6–0.8) were transferred to deoxygenated DM medium establishing an initial OD_600_ of approximately 0.01. Sample aliquots were periodically retrieved, centrifuged at 8,000 rpm for 5 min, and the supernatants were subsequently filtered through a 0.45 μm sterile filter membrane, then stored at 4°C. Standard solutions were prepared as follows: a gradient of SCFAs (acetic, propionic, isobutyric, butyric, isovaleric, and valeric acids) at concentrations ranging from 0.05 to 0.40 g/L, and a 1 g/L mixture including acetic acid, succinic acid, pyruvate, lactic acid, and 1,2-propanediol. To all standards, 10 μL of 4-methylvalerate (12 g/L) was added as an internal standard. Additionally, 10 μL of 0.06 M HCl was included to enhance chromatographic separation and stability. These standards were stored at 4°C.

The SCFAs were quantified using a gas chromatograph (Agilent 6,890N) equipped with an HP-INNOWax capillary column (30 m × 250 μm × 0.25 μm; Agilent 19,091 N-133I). The instrument settings included an inlet and detector temperatures of 250°C and 260°C, respectively, a nitrogen carrier gas flow of 10.3 mL/min, and an initial column temperature of 140°C for 5 min, increasing by 15°C/min to 180°C, and maintained for 5 min. For other metabolites, similar conditions were used, except the final temperature was increased to 260°C and held for 8 min. The analysis based on retention time and peak area generated SCFA standard curves, and enabled concentration calculations of the sample SCFAs. Measurements were performed in triplicate.

### Analysis of total carbohydrate content

Total carbohydrate content was assessed using the phenol-sulfuric acid method (Masuko et al., 2005) applied to the same batch of samples from the GC analysis. Standards of d-glucose or l-rhamnose, ranging from 0 to 0.10 g/L, matched the dilution of the samples in deionized water. These were mixed in a 2:1:5 ratio with 6% phenol and concentrated sulfuric acid, heated for 5 min in a water bath, then cooled. Absorbance at 490 nm was measured across three replicates, using a 200 μL sample in a 96-well plate, to generate a standard curve and calculate total sugars.

### Spectrophotometric quantification of the rate of intracellular H_2_O_2_ production

The H_2_O_2_ production rate by *B. thetaiotaomicron* cells was quantitatively measured using the Amplex Red (AR) method, which detects resorufin produced from the interaction between AR and H_2_O_2_ catalyzed by horseradish peroxidase (HRP). Resorufin strongly absorbs at 572 nm with a molar absorption coefficient of 58,000 ± 5,000 cm^−1^ M^−1^(Messner and Imlay, 2002; Tian et al., 2021).

A standard curve correlating A_572_ with H_2_O_2_ levels was created. *B. thetaiotaomicron* cells in log phase were centrifuged, resuspended in deoxygenated K_2_HPO_4_ buffer containing monosaccharides to an OD_600_ of 0.01, and incubated at 37°C in air. Cell suspensions were sampled every 3 min, centrifuged to remove cells, and the resulting supernatant was mixed with AR and HRP, and analyzed for A_572_ to calculate H_2_O_2_ concentrations from the established curve.

### Analysis of the antibacterial effect of H_2_O_2_ by plate diffusion method

Defined medium supplemented with glucose and rhamnose (respectively abbreviated as DMG and DMR) agar plates (1.5% agar) were prepared and deoxygenated overnight in an anaerobic chamber. *B. thetaiotaomicron* cells grown in BHI to logarithmic phase (OD_600_ = 0.6–0.8) were centrifuged at 8,000 rpm for 5 min, resuspended in a concentrated form, and 150 μL was spread on each anoxic plate. On a DM plate, two sterile 6 mm disks received 6 μL of sterile water and 1 M H_2_O_2_, respectively. These disks created a gradient of H_2_O_2_ concentration, highest at the center and decreasing with distance, which correspondingly reduced its inhibitory effects on bacterial growth. After 72 h of anaerobic incubation, inhibition zones were measured using a vernier caliper.

### Examination of growth recovery following exposure to air

The experiment assessed *B. thetaiotaomicron* growth and adaptability by incubating cells in anaerobic BHI until reaching mid-log phase (OD_600_ = 0.6–0.8). These were then transferred into deoxygenated DMG and DMR media with an initial OD_600_ of ~0.02 and cultured anaerobically until OD_600_ = 0.1, spanning 3–4 generations. Cells were centrifuged at 8,000 rpm for 5 min, resuspended in oxygenated DM, and shaken at 37°C and 200 rpm for 6 h aerobically. Post-incubation, cells were again centrifuged, resuspended in deoxygenated DM, and cultured anaerobically at 37°C. OD_600_ was recorded periodically throughout the anaerobic-aerobic-anaerobic transition to plot growth curves and calculate generation times before and after oxygen exposure.

### Development of strains overexpressing *rhaR*

The *B. thetaiotaomicron* genome was extracted using a genome extraction kit (Tiangen Biotech.). The *rhaR* gene (BT_3768) and its 450 bp upstream sequence (as promoter) were PCR-amplified with primers XS03 and XS04 (Supplementary Table S2). The PCR product and the vector pNLY1-PsusA were digested with *Bam*H I and *Sac* I, and then ligated using a rapid DNA linking kit (Beyotime Biotech.). The resultant plasmid, p*rhaR*, was transformed into *E. coli* DH5α using the CaCl_2_ method. Positive clones were identified via colony PCR, and the plasmid was isolated from selected clones, verified by restriction analysis, and subsequently introduced into *E. coli* S17-1. The plasmids were then transferred by conjugation from *E. coli* S17-1 to wild-type *B. thetaiotaomicron* and the mutant strain SM136, and positive clones carrying the p*rhaR* plasmid were selected with gentamicin and chloramphenicol.

### Evaluation of gene expression by qRT-PCR

Cells were cultured to logarithmic phase in BHI under anaerobic conditions, then inoculated into deoxygenated DMG and DMR at an initial OD600 of ~0.01. Growth continued to OD600 = 0.3, followed by immediate chilling, centrifugation at 8,000 rpm for 5 min at 4°C, and cell quenching in liquid nitrogen. Remaining cultures were aerated and shaken for 1.5 h before a repeat of the same sample collection process. Samples were stored at −80°C.

RNA extraction involved resuspending cells in 100 μL of 10 mg/mL lysozyme, homogenizing, and using a total RNA isolation kit (Vazyme RC112). Post-electrophoresis quality checks (1% agar) and DNase (Vazyme R323) treatment, cDNA was synthesized using reverse transcriptase (Vazyme R323). RT-qPCR employed SYBR Green (TOYOBO), standard primers with 16S rRNA as the control, and RT-qPCR was carried out in a fluorescence quantitative PCR instrument (Analytik Jena qTower 3G), running for 40 cycles at 95°C for 5 s and 61.5°C for 30 s per cycle. Results analyzed using the 2^−ΔΔCt^ method were based on triplicate experiments.

### Survival rates after aerobic exposure

The experiment assessed the viability of *B. thetaiotaomicron* after exposure to an aerobic environment. Initially, *B. thetaiotaomicron* was cultured in BHI to the mid-logarithmic stage and then transferred to anaerobic DMG and DMR media with an initial OD_600_ of approximately 0.01. The bacteria were grown anaerobically for 3–4 generations until reaching an OD_600_ of about 0.1. Following this, the bacterial suspension was centrifuged at ambient temperature at 8,000 rpm for 5 min, and the cells were resuspended in freshly oxygenated DMG or DMR medium. The cells were then incubated in air for 6 h at 37°C with shaking at 200 rpm. Viability was assessed before and after aerobic exposure by diluting the bacterial suspension 10,000-fold with sterile water, plating 100 μL on deoxygenated DMG or DMR agar, and incubating anaerobically at 37°C. Colony counts were performed after 5 days to calculate the survival rate.

### Gene knockout of *rhaR* by CRISPR-Cas12

The method for knocking out the *rhaR* gene was executed by employing the CRISPR-Cas12 system, referencing the procedures detailed in a previous report (Zheng et al., 2022). Firstly, the sgRNA targeting rhaR was crafted using the design tool accessible at https://chopchop.cbu.uib.no/. Subsequently, the double-stranded DNA encoding this sgRNA sequence wa synthesized by Sangon Biotech (Shanghai) Co., Ltd. The necessary plasmid pB025, containing CRISPR-Cas12 expression components, was courteously provided by Zheng et al. (2022). The plasmid containing sgRNA was constructed using pB025 as a template and BT3768-P1/BT3768-P2 (listed in Supplementary Table S3) as primers, linearized, and verified by gel electrophoresis. The upstream and downstream homology arms of targeted gene were prepared by PCR using the primers BT3768-P3/BT3768-P4 and BT3768-P5/BT3768-P6 (Supplementary Table S3). After DpnI digestion and Gibson assembly, the plasmid was introduced into *E. coli* S17-1, selected on ampicillin, and conjugated into *B. thetaiotaomicron*. This strain was cultured with erythromycin and gentamicin, induced with 100 ng/mL anhydrotetracycline (aTc), and assessed for knockout success by PCR. Plasmid curing was confirmed after 10 passages without antibiotics.

### Statistical analysis

Statistical analyses, comprising hypothesis tests and confidence interval calculations, were conducted using GraphPad Prism 9 software. A significance threshold of *p* < 0.05 was set.

## Data Availability

The raw data supporting the conclusions of this article will be made available by the authors, without undue reservation.
