# Supplementary material for: Bacteroides thetaiotaomicron enhances oxidative stress tolerance through rhamnose-dependent mechanisms
Source: Front Microbiol. 2024 Dec 11;15:1505218. doi: 10.3389/fmicb.2024.1505218 (PMC11669328; doi:10.3389/fmicb.2024.1505218)
Supplement: Supplementary file 1 [file Supplementary_file_1.docx]

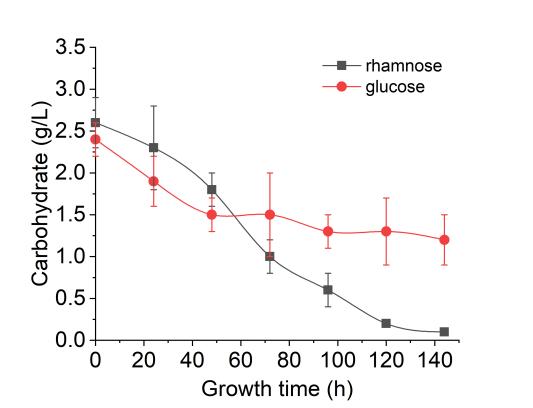


Figure S1. Measurement of monosaccharide carbon source utilization rates. The cells were cultured in DM supplemented with both glucose and rhamnose as carbon sources, carbohydrate content was assessed using the phenol-sulfuric acid method, as described in Materials and methods. Error bars represent the SEM derived from at least three measurements.


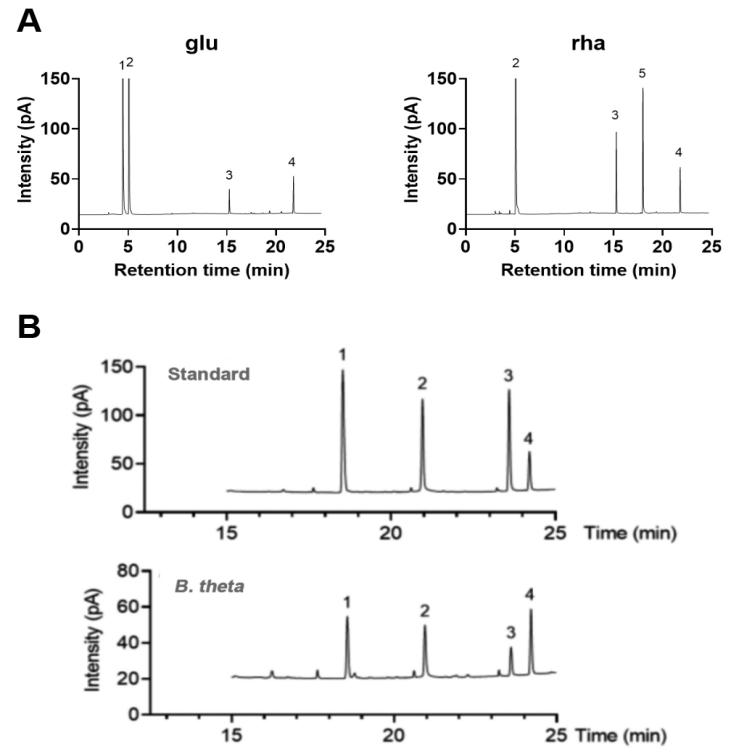


Figure S2. *B. thetaiotaomicron* metabolizes rhamnose to produce 1,2-propanediol and lactic acid. (A) Gas chromatogram of the samples of 6-day group. 1: water; 2: methanol; 3: acetate; 4: 4-methylvaleric acid (interal label); 5: unknown peak. In order to clearly analyze the product peaks, peaks 1 and 2 are not fully displayed. glu, glucose; rha, rhamnose. (B) Standard: retention times of different standards in gas chromatography. 1: acetate; 2: 1,2-propanediol; 3: lactate; 4: 4-methylvaleric acid (internal label). B. theta: peaks of major metabolites after 6 days of growth.


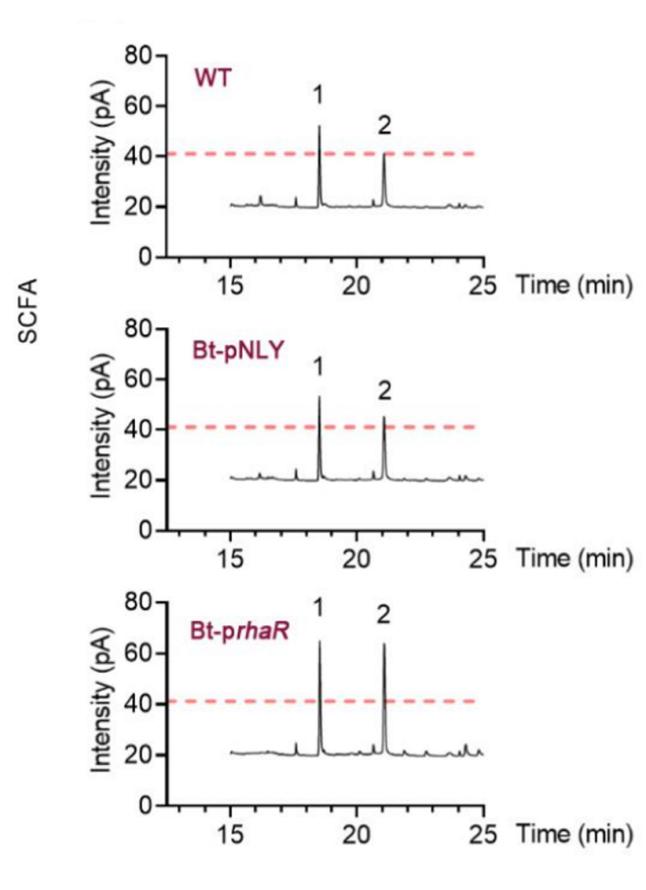


Figure S3. GC spectra of acetic acid (peak 1) and 1,2-propanediol (peak 2) with red dashed lines indicating peak levels of 1,2-propanediol in WT *B. thetaiotaomicron.*


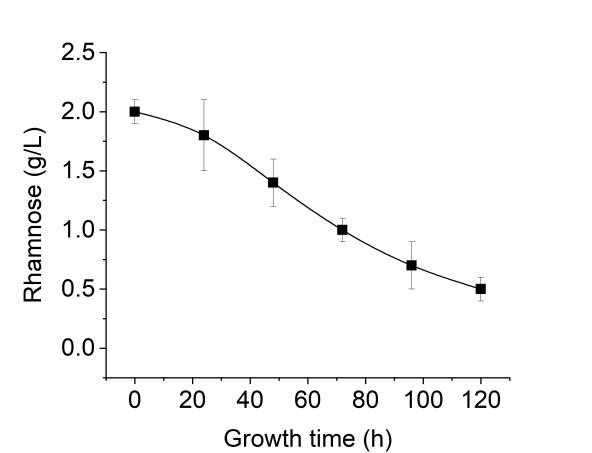


Figure S4. Rhamnose consumption of Δ*rhaR* mutant (JZ005). The rhamnose concentration in the DM media was measured using the phenol-sulfuric acid method, as described in Materials and methods.


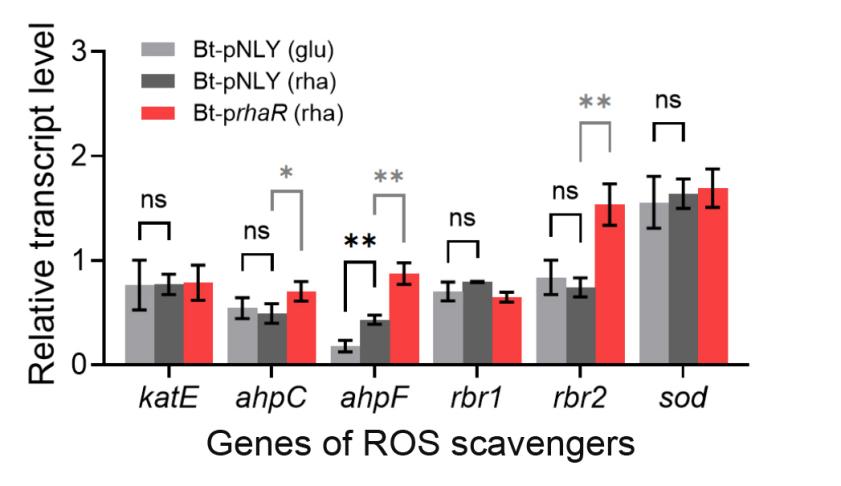


Figure S5. The metabolism of rhamnose does not affect the transcription of most ROS-detoxifying enzymes. The relative transcription level is defined as the transcription level of cells growing in a defined medium (DMG or DMR) after aeration relative to before aeration, and the value is calculated using the 2^-ΔΔCt^ method. glu, glucose; rha, rhamnose. ns, not significant; *, *P* < 0.05; **, *P* < 0.01.

**Table S1. Strains and plasmids**

| **Strain or plasmid** | **Genotype** | **Source** |
| --- | --- | --- |
| ***B. thetaiotaomicron*** |  |  |
| wild type | VPI-5482 | ^1^ |
| Hpx^−^ | VPI-5482 with deletion of *katE*, *ahpC*, *rbr1*, and *rbr2* | ^2^ |
| *B. theta-*pNLY | VPI-5482 containing pNLY-P*susA* (*Cm*^r^) | this study |
| *rhaR*^(+)^ | VPI-5482 harboring p*rhaR* plasmid (*Cm*^r^) | this study |
| ***E. coli*** |  |  |
| DH5a | *end*A *hsdR*17 *supE*44 *thi-1* *recA1 gyrA96 relA1* ∆ (argF-lacZYA) U 169 φ80dlacZ∆M15 | ^3^ |
| S17-1 | RP4-2 (*Km*::*Tn*7, *Tc*::*Mu*-1), pro-82, λ*pir*, *recA*1, *endA*1, *thiE1, hsdR17, creC510* | ^4^ |
| **plasmid** |  |  |
| pNLY-P*susA* | Derived from shuttle vector pBI143 U30316; containing the *susA* promoter; (*Ap*^r^, *Cm*^r^) | ^5^ |
| p*rhaR* | pNLY-P*susA* containing *rhaR* gene and the 450 bp upstream of *rhaR*; (*Cm*^r^) | this study |

**Table S2. Primers used for PCR or RT-PCR**

| Designation | 5'→3' sequence | Use |
| --- | --- | --- |
| XS03 | GGGGGGAAGCTTCTGTTGTTACCTTATGTCATGGA | *rhaR* in pNLY-P*susA* |
| XS04 | GGGGGGCCCGGGTTATTCTTCCCGACCGAACGAG | *rhaR* in pNLY-P*susA* |
| XS0-1F | ACAGCGAGCGTGGCTTCTCATTC | RT-PCR for *rsmH* |
| XS0-1R | GTTGACGATGTCGGCGGCAGTT | RT-PCR for *rsmH* |
| XS1-1F | GCACAGCGTTATCGTCTCGGAGTG | RT-PCR for *katE* |
| XS1-1R | TGGTTGCACCGTAGTTGCCGTCTA | RT-PCR for *katE* |
| XS2-1F | TTGTGGCTACTCACGACGGAGAAGT | RT-PCR for *ahpC* |
| XS2-1R | CCGACAAGGTCAATGCTTGGCTTCA | RT-PCR for *ahpC* |
| XS3-1F | TCGCCATCCTGAATCTGGACGGTAA | RT-PCR for *ahpF* |
| XS3-1R | CGGACAGTTGGTGCAAGTCAGTGAG | RT-PCR for *ahpF* |
| XS4-1F | CGGCGAACACGAAGAATGGTCACT | RT-PCR for *rbr1* |
| XS4-1R | TGCGGTACATAGCAGCAATCATCGG | RT-PCR for *rbr1* |
| XS5-1F | TCAGGCTGACCGTGAAGGTTATCCT | RT-PCR for *rbr2* |
| XS5-1R | TCCCAAACACAGTCACCCAGCAATT | RT-PCR for *rbr2* |
| XS6-1F | AGTCGGATTGTTCGGTTCAGGATGG | RT-PCR for *sod* |
| XS6-1R | GCGTACAGGGTTACTGCCGTTTCC | RT-PCR for *sod* |
| XSK-1F | TTGGTAGCTCACCGTGGCGAATCTA | RT-PCR for *rhaK* |
| XSK-1R | GCATAAGGCTGGCGAAGCAGGTT | RT-PCR for *rhaK* |
| XSI-1F | TTGCTGGCAGGCTGATGATGTAACG | RT-PCR for *rhaI* |
| XSI-1R | TTCAGAATGTCGGCACGCAGTTCA | RT-PCR for *rhaI* |
| XSP-1F | TTGGCAGGTGGTGTGGATGGACTT | RT-PCR for *rhaP* |
| XSP-1R | GCAGGCAGTAAGCGGCATTTGTCA | RT-PCR for *rhaP* |
| XSA-1F | CTAGTTCGGTAGAGTTGGCGGAAGC | RT-PCR for *rhaA* |
| XSA-1R | GCATAGCATCGCAGTCTACGGCAA | RT-PCR for *rhaA* |
| XSO-1F | CGTAGCTTTGGGTGGTGGTTCTTCT | RT-PCR for *rhaO* |
| XSO-1R | TGGTATCAGCCACACCTTCCAGAGA | RT-PCR for *rhaO* |
| XSR-1F | AAGGTCGGCTGATGGTGTTGTTTCC | RT-PCR for *rhaR* |
| XSR-1R | GCATCGCCCACAATCGTATCAATCG | RT-PCR for *rhaR* |
| XSP-1F | CGCTCCATGTATCAACCACGGTCTG | RT-PCR for *PFOR* |
| XSP-1R | TGTAACGCCACAAGTGCCAGTAACC | RT-PCR for *PFOR* |
| XSP-2F | GCTTCGGTAACGACAACGACAAGGT | RT-PCR for *PFL* |
| XSP-2R | CAAGGAGAGGGTAGGACGGGCATT | RT-PCR for *PFL* |

**Table S3. Primers used for knockout of *rhaR* in *B. thetaiotaomicron***

| **Linearization Primers for pB025 Vector**：  BT3768-P1:  GGAAGGTGATCTTCCGGGGGCTTTCTCATGCGTTGGATCCTTGGCTATCAGTCTATCCCTCCCGAATCTACAAGAGTAGAAATTA  BT3768-P2:  aattccgaacaactggaggctttaaggccgaccgcttgtatgaatccatcaaaattc |
| --- |
| **BT3768 Knockout Primers**  BT3768-P3：  CATGAGAAAGCCCCCGGAAGATCACCTTCCCATCGAAAGTTATATAACTCCGGGTGCATG  BT3768-P4：  **AGTAAGCCCGGTACAAGTAGGAATTATT**TTCAAAAAACACACGC  BT3768-P5：  **AATAATTCCTACTTGTACCGGGCTTACT**CCGCTTGAATACCGCT  BT3768-P6：  cggccttaaagcctccagttgttcggaattAAACAAGCAGGATGTGACTTGTTCACAACC |
| **Primers for the knockout verification of BT3768**  Forward：AGGATCTGGACAGGTGCTCT  Reverse：ACGCTCTGTCATACGTTCCG |

**References**

1. Koropatkin NM, Martens EC, Gordon JI, Smith TJ. Starch catabolism by a prominent human gut symbiont is directed by the recognition of amylose helices. Structure. 2008;16(7):1105-1115. doi:10.1016/j.str.2008.03.017

2. Mishra S, Imlay JA. An anaerobic bacterium, *Bacteroides thetaiotaomicron*, uses a consortium of enzymes to scavenge hydrogen peroxide. Molecular Microbiology. 2013;90(6):1356-1371. doi:10.1111/mmi.12438

3. Green MR, Sambrook J. Molecular Cloning: A Laboratory Manual. 4th ed. Cold Spring Harbor laboratory press; 2012.

4. Simon R, Priefer UB, Pühler A. A broad host range mobilization system for in vivo genetic engineering: transposon mutagenesis in gram negative bacteria. Bio/Technology. 1983;1:784-791. doi:10.1038/nbt1183-784

5. Shipman JA, Cho KH, Siegel HA, Salyers AA. Physiological characterization of SusG, an outer membrane protein essential for starch utilization by *Bacteroides thetaiotaomicron*. Journal of Bacteriology. 1999;181(23):7206-7211. doi:10.1128/jb.181.23.7206-7211.1999
